# Characterization of the full-length transcriptome of female *Eucoleus annulatus* and comparative transcriptomic analysis of its head, middle, and tail body sections

**DOI:** 10.1186/s13071-026-07388-z

**Published:** 2026-04-06

**Authors:** Yi-Dan Wang, Jun-Jun He, Cui-Rong Xu, Lu-Yang Wang, Jian-Fa Yang, Ping Wang, Fan-Fan Shu, Feng-Cai Zou, Jun Ma

**Affiliations:** 1 Faculty of Animal Science and Technology, Yunnan Agricultural University, Kunming, 650201 Yunnan Province People’s Republic of China; 2 The Yunnan Key Laboratory of Veterinary Etiological Biology, College of Veterinary Medicine, Yunnan Agricultural University, Kunming, 650201 Yunnan Province People’s Republic of China; 3 Yuxi Agriculture Vocation-Technical College, Yuxi, 653100 Yunnan Province People’s Republic of China

**Keywords:** *Eucoleus annulatus*, PacBio Iso-Seq, Illumina RNA-Seq, Full-length transcriptome

## Abstract

**Background:**

*Eucoleus annulatus* is a parasitic nematode that inhabits the upper digestive tract of avian hosts, posing significant threats to avian health and poultry production. However, the gene information and gene expression characteristics underlying its physiological specialization and parasitic adaptation remain poorly understood.

**Methods:**

In this study, we applied an integrated transcriptomic approach to generate a high-quality full-length transcriptome of *E. annulatus* using PacBio Iso-Seq and to characterize body section-specific gene expression patterns using Illumina RNA sequencing (RNA-Seq). Differentially expressed transcripts (DETs) among its head, middle, and tail sections were identified, and their functional annotations were assessed through Gene Ontology (GO) and Kyoto Encyclopedia of Genes and Genomes (KEGG) enrichment analyses.

**Results:**

PacBio Iso-Seq generated 21,951 high-confidence, non-redundant full-length transcript isoforms, among which 6921 were annotated in the Nr, Pfam, COG, KEGG, and GO databases. Comparative RNA-Seq analysis revealed pronounced section-specific transcriptional divergence, with 1570, 1533, and 1600 DETs enriched in the head, middle, and tail sections, respectively. DETs in the head were significantly enriched in pathways related to amino acid metabolism, RNA processing, and ion transport, while the DETs in the middle body section were primarily associated with glycolysis, oxidative phosphorylation, and transcriptional regulation, indicating elevated metabolic and transcriptional activity. DETs in the tail were significantly enriched in processes related to protein degradation, structural maintenance, and stress adaptation, suggesting roles in environmental response and physiological resilience.

**Conclusions:**

This study, for the first time, reports the first full-length transcriptome of *E. annulatus* and reveals distinct gene expression profiles across different body sections. These findings provide valuable molecular insights into the spatial organization of gene expression in *E. annulatus* and establish a foundation for studying its biology and host–parasite interactions in the future.

**Graphical Abstract:**

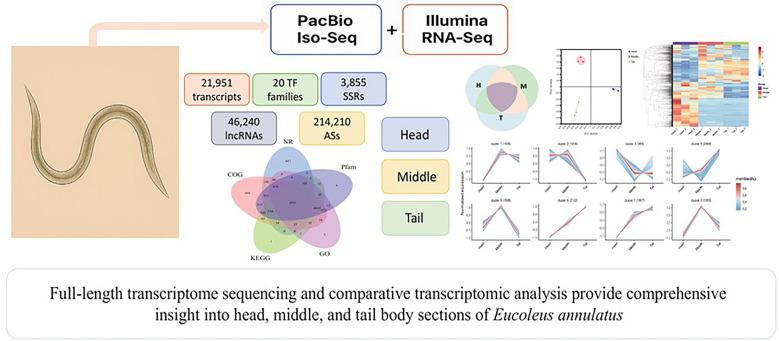

**Supplementary Information:**

The online version contains supplementary material available at 10.1186/s13071-026-07388-z.

## Background

Parasitic nematodes of the family Capillariidae impose substantial health and economic burdens on avian populations worldwide. *Eucoleus annulatus* (syn. *Capillaria annulata*) is of particular concern owing to its global distribution and pronounced tropism for the upper digestive tract of avian hosts [1, 2]. Adult *E. annulatus* inhabit the mucosa of both the esophagus and crop, where infection can induce erosive lesions, granulomatous inflammation, dysphagia, growth retardation, and, in severe cases, mortality, especially in juvenile birds [3]. The life cycle of *E. annulatus* comprises egg, larval, and adult stages. Morphologically, adult *E. annulatus* exhibit pronounced regional differentiation along the body axis, including a slender head region that facilitates tissue penetration and an expanded posterior region in females that is specialized for egg production [4]. Transmission occurs through two main routes, including either direct ingestion of embryonated eggs or indirect infection via earthworm intermediate hosts (*Lumbricus* spp.) [5]. Reported prevalence rates vary widely, ranging from 17.6% in Turkish pheasants (*Phasianus colchicus*) to as high as 98% in birds from irrigated regions of Spain [6, 7]. The parasite is particularly endemic in rural and resource-limited areas, including parts of Africa, where effective prevention and control strategies are often lacking [8–12].

Despite its wide distribution and pathogenic potential, molecular studies on *E. annulatus* remain scarce, largely due to the absence of comprehensive genomic resources. To date, available molecular data are largely restricted to a limited number of mitochondrial genomes, which have provided preliminary phylogenetic insights [13]. The lack of comprehensive genomic and transcriptomic information substantially constrains our understanding of its biology, host–parasite interactions, and mechanisms of pathogenicity [14]. Transcriptome sequencing has been a powerful approach for gene discovery and functional characterization in species lacking a reference genome [15]. In particular, third-generation long-read sequencing technologies, such as PacBio single-molecule real-time (SMRT) sequencing, enable accurate reconstruction of full-length transcripts and offer unique advantages for investigating gene expression in the absence of a reference genome [16]. More recently, integrative strategies combining long-read sequencing (PacBio Iso-Seq) with second-generation short-read sequencing (Illumina RNA-Seq) have proven effective for simultaneously resolving transcript structures and quantifying gene expression in nonmodel organisms without a reference genome [17, 18].

In nematodes, gene expression can vary substantially along the anterior–posterior body axis, reflecting regional functional specialization. However, most transcriptomic studies of parasitic nematodes rely on whole-body samples, which can obscure section-specific expression patterns. Therefore, analyzing transcriptomes from distinct body regions provides a useful framework for linking molecular programs to anatomical specialization and parasitic adaptation. In this study, we applied an integrated transcriptomic strategy combining PacBio Iso-Seq and Illumina RNA-Seq to generate the first full-length transcriptome and to characterize section-specific gene expression profiles across the head, middle, and tail body sections of female *E. annulatus*. This comprehensive dataset provides novel molecular insights into the anatomical specialization of *E. annulatus* and establishes a valuable foundation for future studies on its biology and parasitic adaptation.

## Methods

### Sample collection and RNA extraction

Adult female *E. annulatus* were collected from the upper gastrointestinal tract of green peafowls (*Pavo muticus*), immediately snap-frozen in liquid nitrogen, and stored at −80 °C until further processing. Species identification was confirmed by DNA sequencing of the *cox1* gene. For differential gene expression analysis across anatomical body sections, each female *E. annulatus* was dissected into three equal-length sections (head, middle and tail as illustrated in Supplementary Fig. S1). Total RNA was extracted from the tissue using TRIzol^®^ Reagent (Invitrogen, USA) according to the manufacturer’s instructions. RNA integrity and quality were assessed using an Agilent 5300 Bioanalyzer (Agilent Technologies, USA), and RNA concentration was determined using an ND-2000 spectrophotometer (NanoDrop Technologies). Only high-quality RNA samples meeting the criteria were used for library construction (OD260/280 ratios between 1.8 and 2.2, OD260/230 ≥ 2.0, RNA quality number (RQN) ≥ 7.0, 28S:18S rRNA ratio ≥ 1.0, and total RNA yield > 2 μg).

### PacBio Iso-Seq

For full-length transcriptomic analysis, full-length complementary DNA (cDNA) was synthesized from total RNA extracted from whole *E. annulatus* using the SMARTer™ PCR cDNA Synthesis Kit (Takara Bio, USA) [19]. Libraries were prepared by amplifying, repairing, and ligating cDNA to SMRTbell adapters, followed by assessment on an Agilent 5300. Sequencing was performed on a PacBio Sequel II platform (PacBio, USA). Raw subreads were processed using the Iso-Seq3 pipeline implemented within SMRT Link. Low-quality reads were filtered, and adapter, primer, and sequencing connector sequences were removed to obtain high-quality clean data. Circular consensus sequences (CCSs) were generated from subreads using the following criteria: full passes ≥ 3 and sequence accuracy > 0.9. CCSs were then classified to identify full-length non-chimeric (FLNC) and non-full-length (nFL) reads on the basis of the presence of both 5′ and 3′ primers and a poly(A) tail. FLNC reads were subsequently clustered to generate consensus isoforms, followed by error correction using the iterative clustering for error correction (ICE) algorithm [20]. Consensus sequences were further polished using Quiver to improve base-level accuracy [21]. The resulting polished consensus sequences were used as reference transcripts for downstream analyses. Redundant transcript isoforms were removed using the Iso-Seq3 collapse function on the basis of sequence similarity, and the number and quality of non-redundant transcripts were assessed. Transcriptome completeness was evaluated using Benchmarking Universal Single-Copy Orthologs (BUSCO) version 5 [22] to assess the integrity and coverage of the assembled full-length transcriptome.

### Transcript annotation

Non-redundant FLNC transcripts were submitted to public databases for functional annotation, including the Clusters of Orthologous Groups/Eukaryotic Orthologous Groups (COG/KOG) database (http://www.ncbi.nlm.nih.gov/COG), Gene Ontology (GO) database, National Center for Biotechnology Information (NCBI) non-redundant protein (Nr) database, Kyoto Encyclopedia of Genes and Genomes (KEGG) database (http://www.genome.jp/kegg), evolutionary genealogy of genes (eggNOG) database (http://eggnog5.embl.de/), and Pfam database (https://pfam.xfam.org/). Putative long noncoding RNAs (lncRNAs) were predicted via the integration of results from four computational tools, including CPC2 [23], CNCI [24], the Pfam protein family (Pfam) database (https://pfam.xfam.org), and PLEK [25]. Transcripts longer than 200 nucleotides with coding potential scores below the thresholds across all tools were retained as high-confidence lncRNAs. Transcription factors (TFs) were identified by using AnimalTFDB version 4.0. Simple sequence repeats (SSRs) were detected from the full-length transcripts using MISA, with default parameters for mono- to hexanucleotide repeat motifs. Alternative splicing (AS) events were identified from the Iso-Seq data using SUPPA2 and classified into common types, including exon skipping, intron retention, and alternative donor or acceptor sites.

### RNA-Seq and data analysis

Total RNA from head (H), middle (M), and tail (T) body sections of adult female *E. annulatus* (three biological replicates per section) was sequenced on the Illumina HiSeq platform (PE150). The raw reads were trimmed using Cutadapt version 1.9 to remove adapters and low-quality bases (*Q* < 20). Clean reads were aligned to the PacBio Iso-Seq-derived reference transcriptome using HISAT2 version 2.2.1 with default parameters. Aligned reads were assembled and quantified at the transcript level using StringTie version 2.1.4. Raw read count matrices were used as input for differential expression analysis. Differentially expressed transcripts (DETs) among the head, middle, and tail sections were identified using DESeq2 version 1.34.0 in R, with thresholds set at |log_2_ fold change|≥ 1 and a false discovery rate (FDR) < 0.05. For expression visualization and exploratory analyses, transcript abundance was normalized as fragments per kilobase of transcript per million mapped reads (FPKM). Normalized FPKM values were used for principal component analysis (PCA). For hierarchical clustering and heatmap visualization, expression values were log-transformed as log_2_FPKM. Spatial expression clustering across anatomical sections was conducted using the Mfuzz package version 2.52.0 [26] in R. Gene Ontology (GO) [27] and Kyoto Encyclopedia of Genes and Genomes (KEGG) [28] enrichment analyses were performed using the clusterProfiler package version 4.2.2.

### Verification of RNA-Seq data by quantitative real-time PCR

The RNA samples used for the construction of the RNA-Seq library were reverse transcribed into cDNA using the TransScript^®^ Uni All-in-One First-Strand cDNA Synthesis SuperMix for qPCR Kit (TransGen Biotech, Beijing, China). Ten *E. annulatus* transcripts that were randomly selected to validate RNA-Seq results by quantitative real-time polymerase chain reaction (qRT-PCR), with reduced glyceraldehyde 3-phosphate dehydrogenase (GAPDH) used as an internal reference gene. Primers sequences used for qRT-PCR are listed in Supplementary Table S1. qRT-PCR was performed with PerfectStart^®^ Green qPCR SuperMix (TransGen Biotech, Beijing, China) on a Bio-Rad CFX96 system. The amplification program consisted of an initial denaturation at 94 °C for 30 s, followed by 40 cycles of denaturation at 94 °C for 5 s and annealing at 60 °C for 30 s. Melting curve analysis ranged from 65 to 95 °C to confirm the specificity of amplification. Relative transcript expression levels were calculated using the 2^−ΔΔCt^ method [29].

## Results

### Full-length transcriptome assembly and annotation of *E. annulatus*

**Fig. 1:**
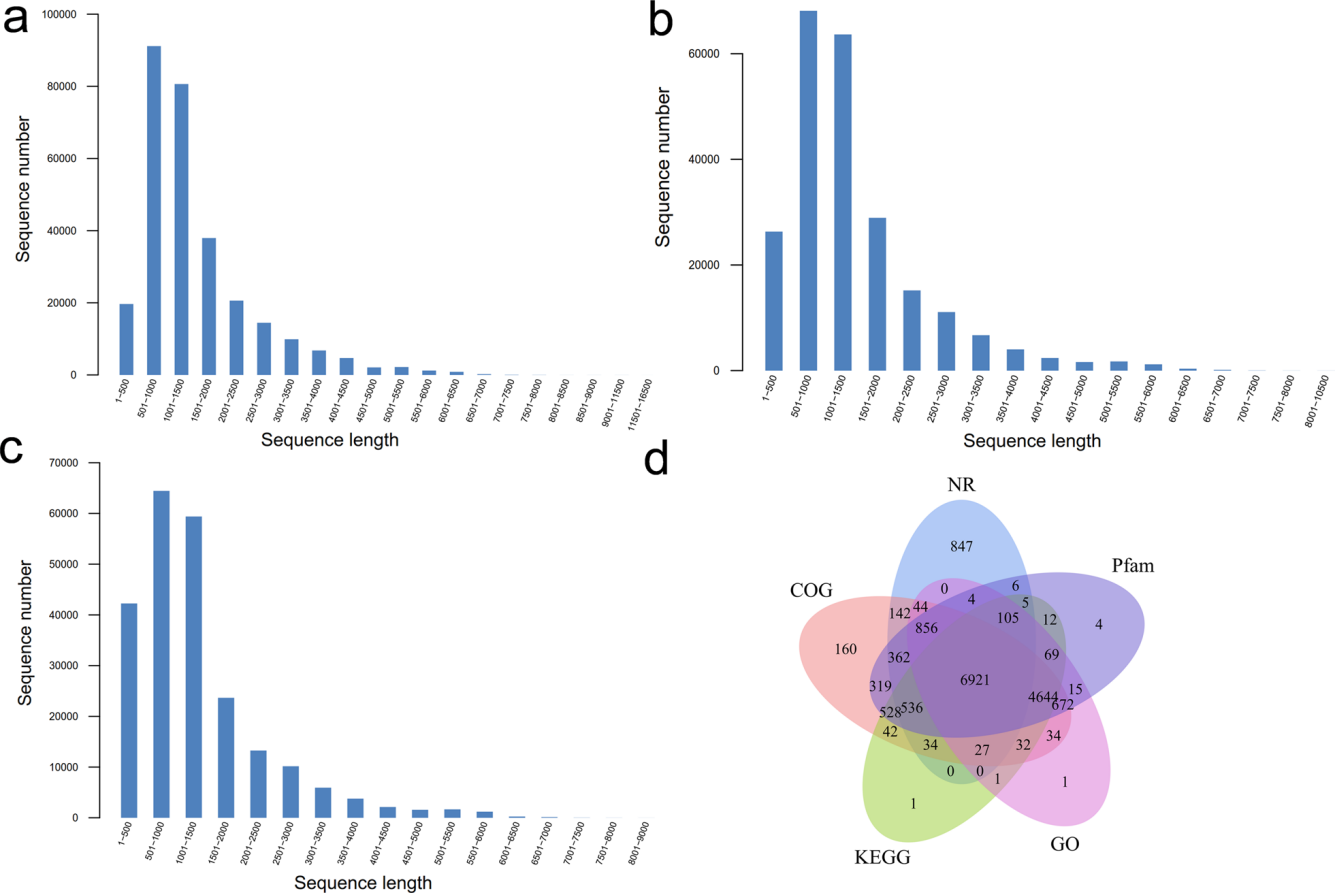
Overview of PacBio SMRT sequencing of *E. annulatus*. **a** Length and quantity distributions of 292,579 circular consensus sequence (CCS) reads. **b** Length and quantity distributions of 231,557 full-length (FL) reads. **c** Length and quantity distributions of 229,875 full-length nonchimeric (FLNC) reads. **d** Venn diagram showing the overlap of annotated transcripts in the Nr, Pfam, COG, KEGG, and GO databases. A total of 6921 transcripts were annotated across all five databases

To generate a comprehensive full-length dataset for adult female *E. annulatus*, high-quality total RNA was extracted and sequenced using the PacBio Sequel II platform. In total, 24.90 Gb of subread data were generated, yielding 21,226,543 filtered subreads with an average read length of 1172.9 bp. From these data, 292,579 circular consensus sequences (CCSs) with lengths predominantly ranging from approximately 500 to 1500 bp were obtained (Fig. 1a). After quality filtering, 231,557 full-length (FL) reads were obtained (Fig. 1b), including 229,875 full-length non-chimeric (FLNC) reads (Fig. 1c). Subsequent error correction and redundancy removal using CD-HIT resulted 21,951 high-confidence, non-redundant transcript isoforms. Functional annotation against five major public databases (Nr, Pfam, COG, KEGG, and GO) showed that 9889, 15,058, 15,353, 12,957, and 13,426 transcripts were annotated in each database, respectively. Among these, 6921 transcripts were common to all five databases (Fig. 1d; Supplementary Table S2). Notably, a substantial proportion of transcripts remained unannotated, indicating the presence of potentially novel or species-specific genes in *E. annulatus*. Transcriptome completeness was further assessed using BUSCO version 5. Among the 255 BUSCO groups searched, 61.5% were identified as complete, including 38.4% single-copy and 23.1% duplicated BUSCOs, while 18.8% were fragmented and 19.7% were missing (Supplementary Table S3). Given that this dataset represents a stage- and sex-specific transcriptome rather than a complete genome assembly, these results indicate a moderate but expected level of completeness for the *E. annulatus* full-length transcriptome.

### Characterization of TFs, SSRs, AS events, and lncRNAs

**Fig. 2:**
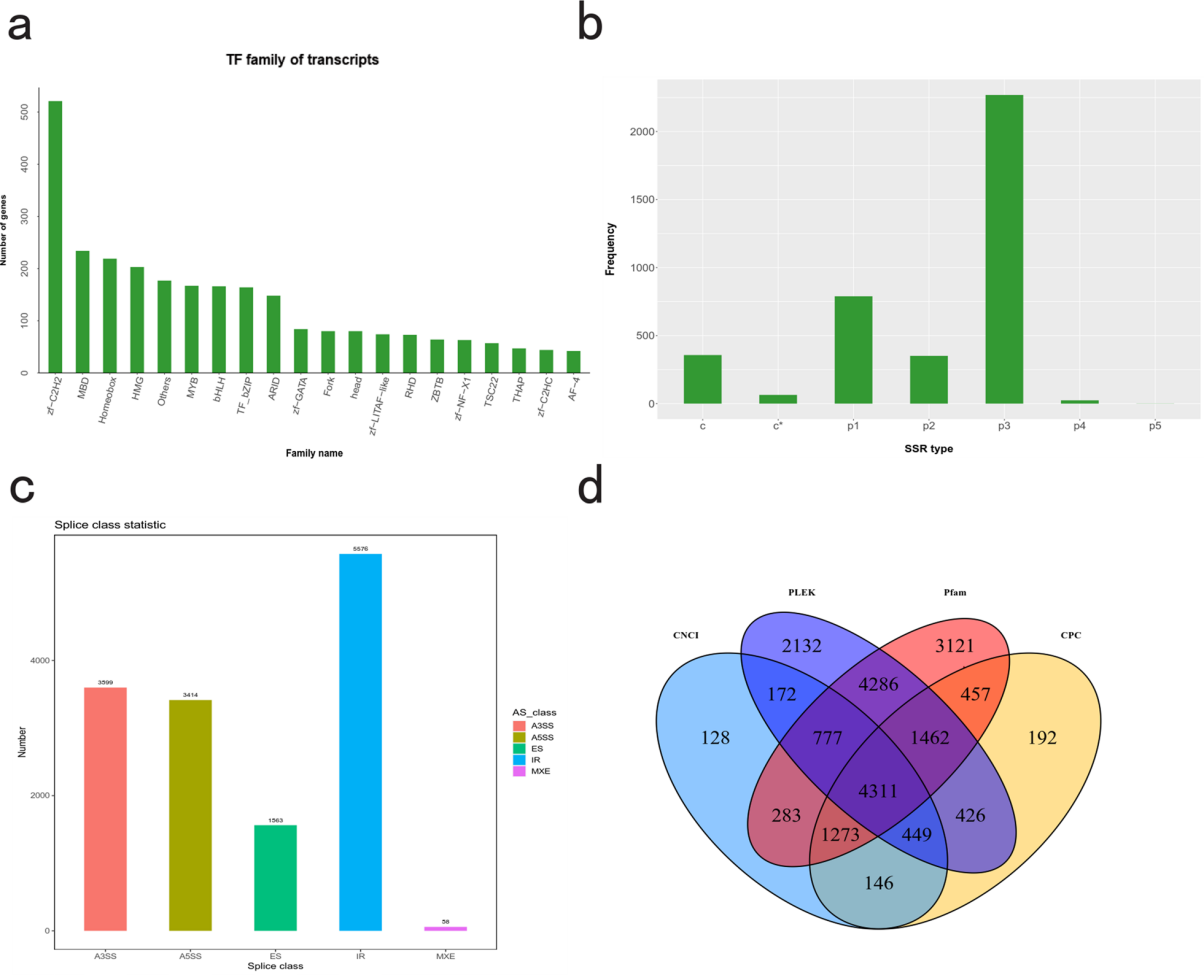
Full-length transcriptomic analysis of *E. annulatus.* **a** Distribution of identified transcription factor (TF) families. **b** Statistics and distribution of simple sequence repeats (SSRs) identified in the transcriptome. **c** Types of alternative splicing (AS) events detected. **d** Venn diagram showing the number of shared and unique lncRNAs predicted via the CNCI, PLEK, Pfam, and CPC2 tools. (c = compound SSRs; c* = compound (with interruption); p1–p5 = mono-, di-, tri-, tetra-, and pentanucleotide repeats)

To further characterize the regulatory and transcriptomic landscape of *E. annulatus*, transcription factors (TFs), simple sequence repeats (SSRs), alternative splicing (AS) events, and long noncoding RNAs (lncRNAs) were systematically analyzed. In total, 20 distinct TF families were identified, with the zf-C2H2 family representing the most abundant groups. In contrast, the AF-4 family contained the fewest TF members (Fig. 2a; Supplementary Table S4). A total of 3855 SSRs were detected, among which trinucleotide repeats constituted the largest proportion, accounting for 58.86% of all SSRs (Fig. 2b). Overall, 14,210 AS events were identified across the transcriptome. Intron retention (IR) was the most prevalent AS type, with 5576 events, whereas mutually exclusive exons (MXEs) were the least frequent (Fig. 2c). Furthermore, a total of 46,240 lncRNA candidates were initially predicted using individual computational approaches; after integrating results from CPC2, CNCI, Pfam, and PLEK, 4311 lncRNAs consistently classified as noncoding by all methods were retained as high-confidence lncRNAs, indicating that lncRNAs constitute a substantial component of the adult female *E. annulatus* transcriptome (Fig. 2d; Supplementary Table S5).

### Comparative transcriptomic profiling of head, middle, and tail body sections

**Fig. 3:**
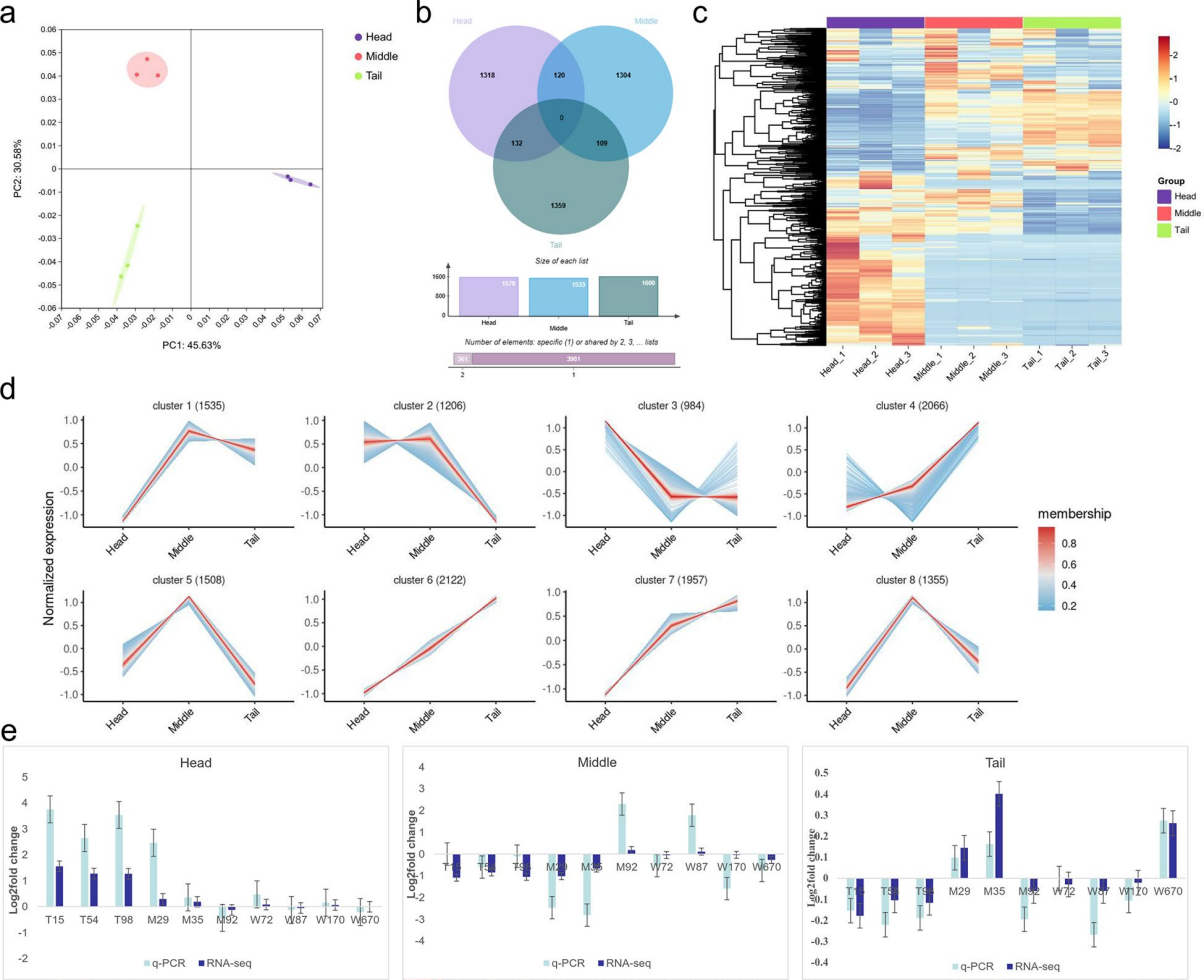
Transcriptomic comparison across head, middle, and tail sections of *E. annulatus*. **a** Principal component analysis (PCA) based on FPKM values, showing distinct clustering of transcriptomes from the head (H), middle (M), and tail (T) sections. **b** Venn diagram showing the overlap and specificity of DETs identified in pairwise comparisons among H, M, and T. **c** Hierarchical clustering heatmap illustrating DETs expression patterns across H, M, and T. Each row represents a gene, and each column represents a biological replicate. Expression values were calculated as log_2_ FPKM and normalized by row-wise *Z*-score transformation; the color scale represents relative expression levels ranging from −2 to 2. **d** Transcripts were clustered into eight groups via fuzzy c-means clustering (Mfuzz) on the basis of normalized expression profiles across the head, middle and tail anatomical sections. **e** Relative expression levels of ten selected DETs validated by qRT-PCR and RNA-Seq. The *x*-axis indicates gene IDs, and the *y*-axis shows relative expression levels

To characterize transcriptomic differences among different anatomical sections of *E. annulatus*, RNA sequencing was performed on the head (H), middle (M), and tail (T) sections using the Illumina HiSeq Ten X platform. A total of nine RNA libraries were constructed (three biological replicates per section), generating more than 188.81 million raw paired-end reads. After quality filtering, 56.24 Gb of clean data were retained. The Q30 scores ranged from 92.82% to 94.85%, and the GC content varied between 48.30 and 51.58%, indicating high sequencing quality (Supplementary Table S6). Clean reads were aligned to the PacBio Iso-Seq-derived reference transcriptome, and transcript abundance was quantified using the FPKM method. PCA based on normalized expression levels showed clear separation among the three anatomical sections, with tight clustering of biological replicates within each section. Notably, head and tail samples exhibited the greatest separation along the first principal component (PC1) (Fig. 3a). These results demonstrate distinct transcriptomic profiles across different body sections of *E. annulatus*.

### Differentially expressed transcripts and clustering analysis

Compared with the other anatomical sections, a total of 1570, 1533, and 1600 DETs were identified in the head, middle, and tail body sections, respectively (Supplementary Tables S7–9). As illustrated by the Venn diagram (Fig. 3b), no DETs were shared among the three section-specific comparisons. Consistently, heatmap analysis showed that DETs from different anatomical sections formed distinct expression patterns and clustered into clearly separated modules (Fig. 3c).

To further characterize section-specific transcriptional organization in adult female *E. annulatus*, transcript-level expression profiles from the head, middle, and tail sections were normalized and subjected to soft clustering analysis using Mfuzz (Fig. 3d). In total, 13,733 transcripts were grouped into eight distinct clusters (C1–C8), each representing a characteristic section-associated expression pattern. Clusters 5 and 8, comprising 1508 and 1355 transcripts, respectively, displayed pronounced enrichment in the middle section. Clusters 1 and 2 (1535 and 1206 transcripts, respectively) also showed biased expression toward the middle section, although with more moderate clustering strength. Cluster 3, consisting of 984 transcripts, exhibited head-enriched expression, with transcript abundance peaking in the head and gradually decreasing toward the tail. In contrast, clusters 4, 6, and 7 (2066, 2122, and 1957 transcripts, respectively) were enriched in the tail section and were characterized by progressively increasing expression from head to tail.

**Fig. 4:**
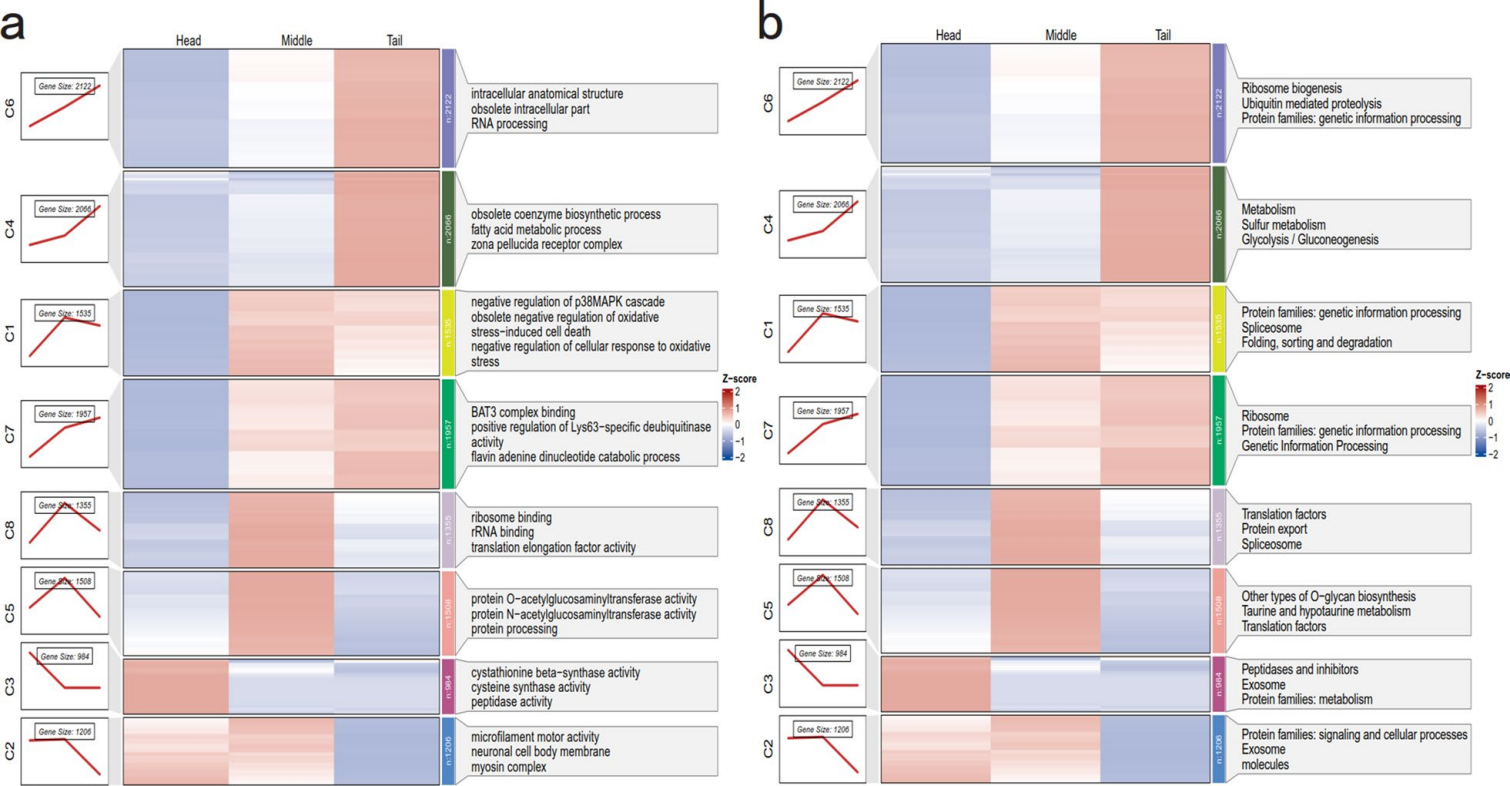
GO and KEGG pathway enrichment of Mfuzz anatomical clusters in *E. annulatus*. Heatmaps display the normalized *Z*-score expression profiles of transcripts grouped into eight Mfuzz clusters (C1-C8) across the head, middle, and tail sections. **a** The top enriched Gene Ontology (GO) terms (top 3 terms ranked by adjusted *P* value) for each cluster are shown to highlight representative biological processes and molecular functions. **b** The top enriched Kyoto Encyclopedia of Genes and Genomes (KEGG) pathways (top 3 pathways ranked by adjusted *P* value) associated with each cluster

As shown in Fig. 4, GO enrichment analysis revealed distinct functional associations among the different expression clusters. Cluster 3 (head-enriched) was significantly associated with metabolic processes, proteolytic regulation, and signal modulation. Middle-enriched clusters, such as C1 and C5, were primarily associated with the oxidative stress response, RNA splicing, and ribosome biogenesis. Tail-enriched clusters, including C4 and C6, were mainly linked to ubiquitin-mediated proteolysis, lipid metabolism, and membrane organization, highlighting functional differentiation along the anterior–posterior body axis (Fig. 4a).

KEGG pathway enrichment analysis further supported these section-associated transcriptional patterns (Fig. 4b). Cluster 3 was significantly enriched in pathways related to peptidase activity, the exosome pathway, and amino acid metabolism (e.g., glycine, serine, and threonine metabolism). Middle-enriched clusters were associated with pathways such as spliceosome assembly, protein processing in the endoplasmic reticulum, and glycolysis/gluconeogenesis. In contrast, tail-enriched clusters showed enrichment in axon guidance, lysosome function, and fatty acid degradation pathways.

### Validation of RNA-Seq data by qRT-PCR

To validate the RNA-Seq results, ten DETs (T15, T54, T98, M29, M35, M92, W72, W87, W170, and W670) were randomly selected for qRT-PCR analysis. The RNA samples used for qRT-PCR were identical to those employed for transcriptomic analyses of the head, middle, and tail sections. The *E. annulatus* GAPDH gene was used as an internal reference for expression normalization. The qRT-PCR results showed expression patterns that were consistent with those obtained from RNA-Seq analysis, supporting the reliability of the transcriptomic data (Fig. 3e).

### Differentially expressed transcripts and functional enrichment analysis

To obtain deeper insight into the functional divergence among the head, middle, and tail section of *E. annulatus*, comprehensive functional enrichment analyses of DETs was carried out using both the Gene Ontology (GO) and Kyoto Encyclopedia of Genes and Genomes (KEGG) databases.

**Fig. 5:**
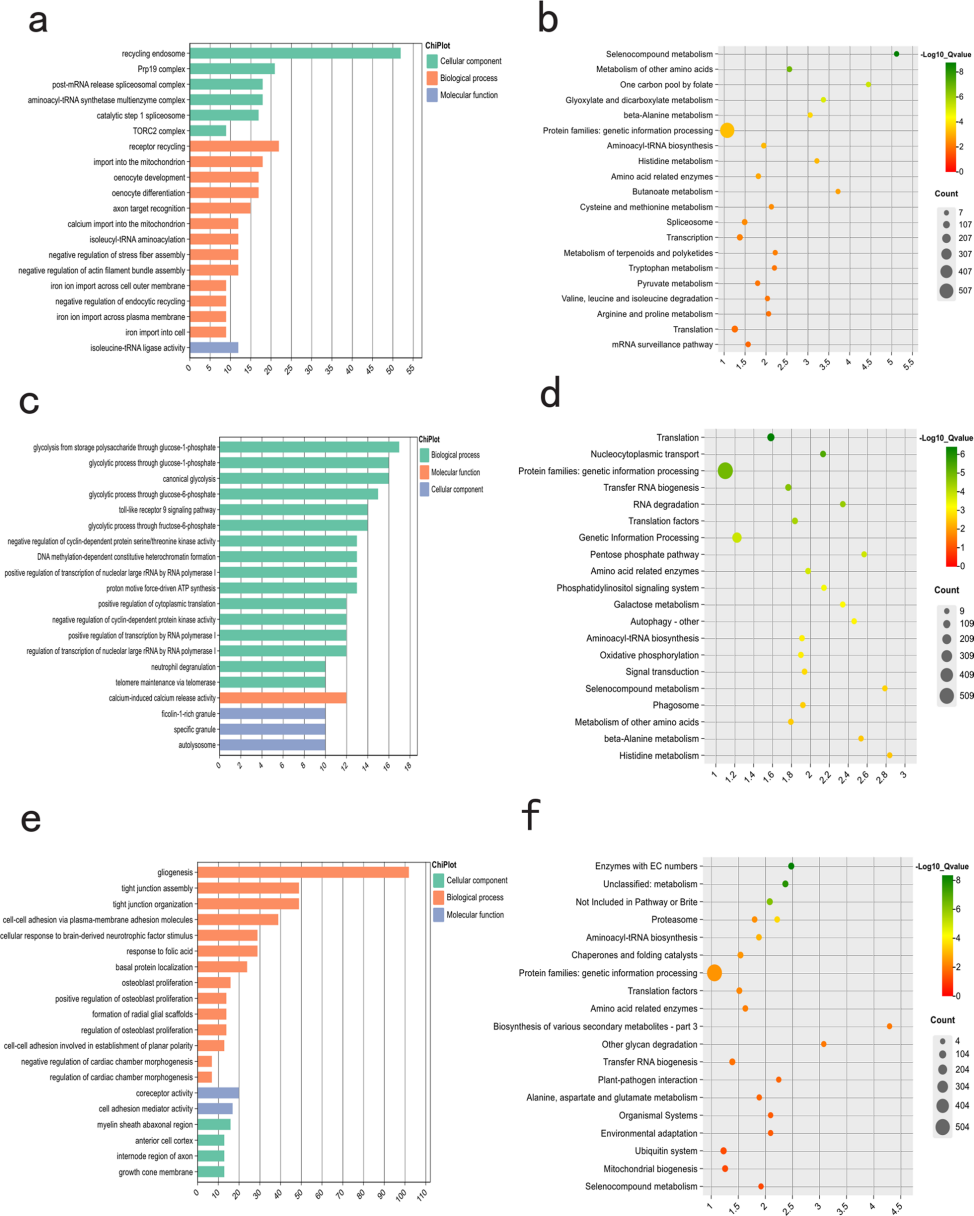
Functional enrichment analysis of DETs in the head, middle, and tail sections of *E. annulatus*. **a** GO enrichment analysis of DETs from head body part GO terms were classified into biological process, molecular function, and cellular component categories. **b** KEGG pathway enrichment analysis of DETs in the head body section. **c** GO enrichment analysis of DETs in the middle body section. **d** KEGG pathway enrichment analysis of DETs in the middle body section. **e** GO enrichment analysis of DETs in the tail body section. **f** KEGG pathway enrichment analysis of DETs in the tail body section

In the head section, DETs were predominantly enriched in GO terms related to RNA processing, receptor recycling, and ion transport (Fig. 5a), including Prp19 complex, catalytic step 1 spliceosome, mRNA surveillance complex, and calcium import into mitochondria. These enrichments indicate prominent post-transcriptional regulation and ion-associated signaling activities in this section. In addition, GO terms such as oenocyte development, axon target recognition, and iron ion import were also significantly enriched, suggesting involvement in neuronal-like activity and active interactions with the host microenvironment. KEGG pathway analysis further revealed significant enrichment in amino acid metabolism and biosynthesis pathways, including histidine metabolism, tryptophan metabolism, and cysteine and methionine metabolism (Fig. 5b). Pathways associated with transcription, translation, the spliceosome, and aminoacyl-tRNA biosynthesis were also prominent. Moreover, enrichment of energy-related pathways, such as glyoxylate and dicarboxylate metabolism and pyruvate metabolism, reflects elevated metabolic activity in the head section.

In the middle section, DETs were significantly enriched in GO terms associated with energy metabolism and gene regulation, including canonical glycolysis, oxidative phosphorylation, DNA methylation-dependent heterochromatin formation, and cytoplasmic translation (Fig. 5c).

GO terms related to Toll-like receptor 9 signaling, neutrophil degranulation, and autolysosome formation were also enriched, indicating potential involvement in host–parasite interaction and immune-related processes. Consistent with these findings, KEGG pathway enrichment analysis revealed overrepresentation of pathways involved in genetic information processing, such as translation, RNA degradation, and nucleocytoplasmic transport, as well as metabolic pathways including the pentose phosphate pathway, galactose metabolism, and oxidative phosphorylation (Fig. 5d). In addition, signal transduction pathways, such as the phosphatidylinositol signaling system, and pathways related to autophagy and phagosome formation were enriched, suggesting roles in cellular regulation and homeostasis.

In the tail section, GO enrichment analysis revealed enrichment with terms related to structural development, adhesion or neural patterning, including tight junction assembly, cell–cell adhesion involved in planar polarity, and formation of radial glial scaffolds (Fig. 5e). Neurodevelopment-related GO terms, such as gliogenesis and axon guidance, as well as reproduction-associated processes, including regulation of osteoblast proliferation, were also enriched, reflecting functional diversity within this section. KEGG pathway analysis showed significant enrichment of DETs in protein degradation and folding pathways, including the proteasome, ubiquitin-mediated proteolysis, and chaperone-related pathways, indicating active protein quality control in the tail section (Fig. 5f).

In addition, pathways involved in aminoacyl-tRNA biosynthesis and translation were enriched, suggesting sustained local protein synthesis. Notably, DETs in the tail section were also enriched in pathways related to organismal systems and environmental adaptation, such as plant–pathogen interaction and mitochondrial biogenesis, which may reflect specialized physiological adaptations associated with posterior body functions.

## Discussion

Comparative transcriptomic analyses have emerged as powerful approaches for investigating the biology of parasitic nematodes and have uncovered numerous genes involved in host–parasite interactions, including those encoding surface proteins and enzymes that facilitate attachment to and penetration of host tissues [30]. *E. annulatus* is a globally prevalent nematode that causes significant pathology in avian hosts; however, its molecular characteristics remain poorly understood, largely owing to the absence of a reference genome. In particular, genes that are preferentially expressed in specific anatomical sections may participate in interactions with host cell surface receptors, potentially triggering downstream signaling pathways and modulating host physiological responses. Despite this, the transcriptional landscape of distinct body sections has not been systematically characterized, which has limited deeper investigations into its biology and parasitic adaptation. In this study, we generated the first full-length transcriptome of *E. annulatus* and performed a comparative transcriptomic analysis across the head, middle, and tail sections of adult female *E. annulatus*. This dataset substantially expands the currently limited molecular resources available for *E. annulatus* and provides a foundation for understanding section-specific transcript expression patterns underlying its anatomical specialization and host interaction.

### Full-length transcriptome reveals transcriptomic complexity

The PacBio Iso-Seq dataset generated in this study provides a comprehensive and high-resolution view of the *E. annulatus* transcriptome, revealing substantial gene structural complexity and extensive isoform diversity. Compared with short-read-based assemblies, third-generation long-read sequencing offers major advantages by capturing transcripts in their complete full-length forms without the need for computational reconstruction [31, 32]. This capability is particularly valuable for nonmodel parasitic nematodes, in which fragmented genomic resources, high gene density, and pervasive alternative splicing frequently compromise accurate transcript assembly using short-read sequencing alone.

Using PacBio Iso-Seq, we identified 21,951 high-confidence full-length transcripts, a substantial proportion of which lacked functional annotation in public databases. Although many transcripts could be assigned putative functions, a large fraction of genes or isoforms remain uncharacterized, highlighting the incomplete representation of nematode transcriptomes in existing databases. This observation is consistent with findings in *Caenorhabditis elegans*, where PacBio Iso-Seq revealed more than 57,000 previously unannotated transcripts derived from 11,921 genes, underscoring the remarkable transcriptomic complexity captured by long-read sequencing [33]. Our results extend this paradigm to the nonmodel species *E. annulatus*, in which transcriptomic complexity may be even greater owing to its parasitic lifestyle and limited genomic resources. Whether these novel or species-specific transcripts contribute to the unique biological adaptations of *E. annulatus* warrants further investigation.

In addition, our analyses identified numerous TFs, SSRs, AS events, and 4311 high-confidence lncRNAs (Fig. 2a–d; Supplementary Tables S4 and S5), reflecting a complex regulatory landscape in *E. annulatus*. Among the TF families, the zf-C2H2 family exhibited the highest transcript abundance (Fig. 2a). This pattern is consistent with studies in the model nematode *C. elegans*, in which C2H2-type zinc-finger proteins constitute one of the largest and most conserved TF families, forming multiple ancestral lineages across bilaterian animals and participating broadly in developmental regulatory networks [34]. In *C. elegans*, C2H2-type TFs have been shown to regulate diverse biological processes, including meiosis (e.g., HIM-8/ZIM family) [35] and neuromuscular function (e.g., UNC-98 and SYD-9) [36]. The prominence of this TF family in *E. annulatus* suggests a potentially conserved role in transcriptional regulation, although functional validation will be required to clarify their specific roles in parasitism and host adaptation.

SSRs are important sources of genetic variability and have been widely applied in population genetic studies of parasitic nematodes [34, 35]. Here, we report the SSR profile of *E. annulatus* for the first time, revealing a predominance of trinucleotide motifs (Fig. 2b). This pattern is consistent with observations in other nematode species. For instance, in *Meloidogyne incognita*, the majority of microsatellites located within coding regions consist of trinucleotide repeats, reflecting functional constraints that favor frame-preserving motifs [37]. Similarly, expressed sequence tag (EST)-derived SSR analyses across multiple plant-parasitic nematodes have demonstrated a dominance of trinucleotide repeats in expressed regions [38]. The prevalence of trinucleotide SSRs in *E. annulatus* therefore suggests a conserved genomic organization across nematode lineages, likely shaped by selective pressures acting on coding sequences. Nevertheless, species-specific variation in SSR motif composition and distribution is common and may reflect divergent evolutionary histories or adaptations to distinct ecological niches [39]. Further comparative analyses across genomic regions and populations will be required to clarify whether SSRs contribute to gene regulation, genome plasticity, or parasitic adaptation in *E. annulatus*.

Alternative splicing (AS) is increasingly recognized as a key mechanism shaping nematode transcriptomes, and the AS landscape identified in *E. annulatus* aligns well with this broader framework. In *C. elegans*, genome-wide analyses have shown that approximately one quarter of protein-coding genes generate multiple isoforms through diverse AS modes, including intron retention (IR) and mutually exclusive exons (MXE), with many events exhibiting developmental or tissue-specific regulation [40]. Mechanistic studies further demonstrate that AS in nematodes is tightly regulated rather than stochastic. For example, Ma et al. showed that the splicing factors UAF-1 and SFA-1 control IR and exon skipping of *tos-1*, providing direct evidence that IR represents a regulated and functionally relevant splice variant rather than a splicing error [41]. Likewise, neuron-specific factors such as the CELF protein UNC-75 regulate sets of cassette and mutually exclusive exons in targets including *unc-32*, highlighting the contribution of exon choice to neuronal subtype-specific functions [42]. A comprehensive screen by Kuroyanagi et al. validated 29 mutually exclusive exon clusters in 27 *C. elegans* genes, underscoring that, although MXE events are relatively infrequent, they are subject to strict regulatory control and are often embedded in genes with specialized functions [43].

Beyond model nematodes, a cross-species survey of nine parasitic species reported alternative splicing in approximately 20–30% of loci, indicating that extensive isoform diversity is a common feature of nematodes with complex life cycles [44]. Although that study did not exhaustively classify all AS event types, subsequent gene-focused investigations have directly linked AS (including exon choice and IR-like events) to traits of pharmacological and biological relevance. In *Haemonchus contortus*, cDNA-amplified fragment length polymorphism (AFLP) analysis revealed alternative splicing of the nicotinic acetylcholine receptor subunit *Hco-acr-8* in levamisole-resistant isolates, implicating AS in shaping drug receptor composition [45]. In filarial nematodes, Kashyap et al. demonstrated that sex-specific isoforms of the SLO-1 potassium channel, generated through alternative splicing within the RCK1 domain, confer differential sensitivity to emodepside in *Brugia malayi* and are conserved in *Onchocerca volvulus*, directly linking AS to anthelmintic efficacy [46]. Together with accumulating transcriptomic and long-read annotations in species such as *H. contortus*, these studies highlight that regulated AS can modulate neuromuscular signaling, host–parasite interactions, and drug responses in parasitic nematodes, rather than being a neutral byproduct of splicing. Within this context, we identified 14,210 AS events in *E. annulatus*, encompassing all five classical splicing types (Fig. 2c). These findings suggest that the AS landscape resolved here, particularly the abundant IR events and selected MXE events, may constitute an additional regulatory layer contributing to section-specific differentiation across the head, middle, and tail, as well as parasitic specialization in *E. annulatus*. Future studies focusing on experimental validation of representative IR and MXE events, assessment of their translational outcomes, and functional characterization during development and host interaction will be essential for deepening our understanding of the molecular biology and parasitic strategies of *E. annulatus*.

Growing evidence shows that lncRNAs are key regulators of transcription, post-transcriptional control, chromatin organization, and genome stability across eukaryotes, including parasites [47, 48]. In nematodes, both long-read and short-read transcriptomic studies have demonstrated that lncRNAs are widely expressed and frequently exhibit stage- or tissue-specific expression. In nematodes, both long-read and short-read transcriptomic studies havedemonstrated that lncRNAs are widely expressed and frequently exhibit stage- or tissue-specific expression. For example, a genome-scale survey in *C. elegans* identified more than 200 lncRNA loci with pronounced developmental regulation, supporting their regulatory potential rather than representing transcriptional noise [49]. In parasitic nematodes, lncRNA catalogs are beginning to emerge. In the blood-feeding *Haemonchus contortus*, whole-transcriptome profiling of the intestine revealed a tissue-resolved atlas of ncRNAs implicated in digestion, cuticle biology, and host-interaction processes [50]. Moreover, differential lncRNA expression programs have been linked to anthelmintic responses; lncRNAs associated with albendazole resistance in *H. contortus* show candidate competitive endogenous RNA (ceRNA) interactions and pathway enrichments relevant to detoxification and development [51]. Long-read approaches in filarial parasites, such as *Brugia malayi* and *Dirofilaria immitis*, have further demonstrated that full-length sequencing improves the resolution of isoforms and operons, thereby enabling more reliable inference of noncoding repertoires and their potential regulatory relationships [52]. In this study, the identification of 4311 high-confidence lncRNAs in *E. annulatus* substantially expands the known repertoire of noncoding regulatory elements in this parasite. Given the established roles of lncRNAs and alternative splicing in nematode development and tissue differentiation, future integrative analyses combining lncRNA expression profiles, alternative splicing patterns, and chromatin-level information may help elucidate how noncoding transcripts contribute to the biological complexity and parasitic adaptation of *E. annulatus*.

### Transcriptomic divergence reflects anatomical specialization and parasitic adaptation

Clear transcriptional divergence among the head, middle, and tail sections of *E. annulatus* indicates pronounced section-specific biological functions. As shown in Fig. 5a, b, DETs enriched in the head section were predominantly associated with recycling endosomes and receptor recycling. Endocytic recycling is a key cellular mechanism that maintains plasma membrane composition and ensures the stable surface abundance of receptors and transporters [53]. In neuronal systems, recycling endosomes are essential for establishing and maintaining membrane polarity, and disruptions in these pathways contribute to a range of neurological disorders [54]. In the context of parasitic nematodes, our results suggest that active membrane protein turnover and receptor recycling represent prominent molecular features of the *E. annulatus* head section. These processes are likely important for sustaining receptor-mediated functions at the anterior end, including sensory perception and environmental responsiveness [55]. Notably, GO terms such as “axon target recognition” likely reflect conserved molecular guidance and signaling pathways rather than evidence for specific neuronal or axonal structures in *E. annulatus*, and thus should be interpreted cautiously. Consistent with this, the enrichment of the GO terms “oenocyte development” and “oenocyte differentiation” was driven by transcripts annotated as Half-A-TPR (HAT) repeat-containing Crooked neck-like protein 1 (CRNKL1) homologs, and most plausibly reflects conserved roles in RNA processing and pre-mRNA splicing rather than insect-specific oenocytes or their differentiation. Therefore, the enrichment of “oenocyte development” and “oenocyte differentiation” in *E. annulatus* does not indicate the presence of insect-specific oenocytes or their differentiation. Instead, it most likely reflects conserved roles of CRNKL1 homologs in RNA processing and developmental or physiological regulation.

The head of nematode plays a central role in host invasion, infection, and immunomodulation. As evidenced by the head-enriched expression pattern of cluster 3 transcripts (Fig. 4a, b; Supplementary Table S10), we observed elevated expression of multiple proteases, including serine proteases, cysteine proteases, as well as their corresponding inhibitors, indicating active proteolytic turnover in the head of *E. annulatus* [56, 57]. Notably, cysteine protease inhibitors secreted by parasitic nematodes have been identified as a major class of immunomodulatory molecules that interfere with host effector mechanisms and facilitate parasite persistence [58]. Trypsin-like serine proteases, which are highly expressed in many parasitic nematodes, are known to participate in host tissue invasion, immune evasion, and nutrient acquisition. For example, *Trichuris muris* secretes serine proteases capable of degrading the mucus barrier component Muc2, thereby promoting persistent infection [59]. In addition, nematode-derived protease inhibitors, such as serpins, are often upregulated during invasive stages and function by inhibiting host serine proteases [60]. This inhibition facilitates epithelial barrier penetration, immune evasion, and parasite establishment, as demonstrated in *Trichuris suis* infections [61, 62]. Together, these observations support the view that the transcriptional specialization of the *E. annulatus* head underpins its role in host interaction and parasitic adaptation.

Compared with the head section, the middle body of *E. annulatus* displayed pronounced enrichment of metabolic and biosynthetic pathways, including glycolysis, oxidative phosphorylation, ribosome biogenesis, and chromatin remodeling (Fig. 5c, d). This pattern is consistent with observations in other parasitic nematodes, in which the central body region functions as a major metabolic hub during active feeding and growth, as reported for *Brugia malayi* [63]. Global expression pattern analysis further revealed that transcripts in cluster 5 (Fig. 4a, b; Supplementary Table S11) were highly expressed in the middle section. Notably, several transcripts encoding O-GlcNAc transferase (OGT) and N-acetylglucosaminyltransferase-related enzymes were strongly enriched in this region (Supplementary Table S11). In *C. elegans*, OGT-1 plays critical roles in nutrient sensing, stress responses, and neuronal signaling [64], suggesting that similar glycosylation-dependent regulatory mechanisms may contribute to metabolic homeostasis in *E. annulatus*. In addition, GO terms related to neutrophil degranulation, Toll-like receptor 9 signaling, and immune regulation were enriched in the middle section. Although nematodes lack a canonical immune system, parasitic helminths are well known to modulate host immunity through secreted effector molecules [65]. Therefore, the immune-associated enrichments observed here likely reflect transcriptional programs involved in immune evasion or host–parasite interactions.

The tail section of *E. annulatus* exhibited a distinct transcriptional profile characterized by enrichment of biological processes related to cytoskeletal organization, cell–cell adhesion, planar polarity establishment, and membrane-associated structures, including terms such as the myelin sheath abaxonal region and growth cone membrane. These GO annotations suggest that the posterior section requires precise regulation of cytoskeletal dynamics and membrane architecture. Enrichment of processes such as tight junction assembly, axonal region organization, and growth cone membrane components may reflect conserved molecular modules supporting cell shape maintenance, polarized structure formation, and localized signal regulation within the tail. Similarly, GO terms related to gliogenesis, radial glial scaffold formation, and basal protein localization are best interpreted as conserved developmental or structural pathways rather than evidence for vertebrate-like tissues. Their presence indicates that transcriptional programs associated with cellular remodeling, polarized transport, and scaffold formation remain active in the posterior section of *E. annulatus*. Such processes may contribute to maintaining structural integrity or supporting localized sensory or regulatory functions, as reported in *C. elegans* [66]. KEGG enrichment analysis further supported a role of the tail section in cellular maintenance and elevated metabolic activity. The tail showed significant enrichment in pathways associated with the ubiquitin–proteasome system, aminoacyl-tRNA biosynthesis, translation factors, chaperones and folding catalysts, and mitochondrial biogenesis. Collectively, these pathways indicate active protein synthesis, degradation, and quality control. Elevated activity of ubiquitin-mediated proteolysis and proteasome components suggests enhanced protein turnover, which may support tissue maintenance or localized stress responses. Enrichment of aminoacyl-tRNA synthetases and translation machinery further implies sustained protein biosynthesis, while mitochondrial biogenesis pathways are consistent with increased energy demand in the posterior section. Consistent with these observations, transcripts in cluster 6 (Fig. 4a, b; Supplementary Table S12) were enriched in functions related to translation, protein folding, and ubiquitin-mediated proteolysis. Comparable posterior-biased expression signatures have also been reported in *Brugia malayi* [63], supporting the conservation of posterior specialization across parasitic nematodes. Notably, the tail-enriched transcriptional program also included transcripts potentially involved in female-specific reproductive regulation. Among these, two transcripts from cluster 7 (Supplementary Table S12), EGJ1500222_transcript_26292 and EGJ1500222_transcript_23137, were annotated as FBXW7-like proteins, a conserved class of F-box E3 ubiquitin ligases. In *C. elegans*, the FBXW7 homolog SEL-10 is known to promote female development and participate in sex determination by targeting intracellular domains of LIN-12/Notch proteins for ubiquitin-dependent degradation [67]. FBXW7 homologs in model nematodes have further been implicated in diverse biological processes, including sex determination, female development, egg-laying behavior, and cell-cycle regulation [68]. The pronounced tail-specific expression of FBXW7-like transcripts in adult female *E. annulatus* therefore suggests that conserved ubiquitin-mediated regulatory mechanisms may contribute to posterior reproductive functions, such as oogenesis, egg maturation, or egg-laying-associated processes. Although functional validation will be required, these findings provide molecular support for the specialization of the tail section in female reproductive biology. Together, these results indicate that the posterior body section of *E. annulatus* possesses distinct physiological specializations related to structural maintenance and local regulatory processes. Further spatially resolved and functional studies will be necessary to verify and refine these interpretations.

## Conclusions

In this study, we integrated long-read PacBio Iso-Seq and short-read Illumina RNA-Seq technologies to generate the first comprehensive full-length transcriptomic profile of adult female *E. annulatus*. Using Iso-Seq, we identified 21,951 high-confidence full-length transcripts and defined a set of 4311 high-confidence lncRNAs through integrative prediction, substantially expanding the currently limited molecular resources available for this undercharacterized parasitic nematode. Differential expression analysis revealed pronounced section-specific transcriptional patterns, reflecting marked anatomical and functional specialization along the anterior–posterior axis. Functional enrichment analyses of DETs by using GO and KEGG further delineated the differential biological characters of head, middle, and tail body sections of *E. annulatus*. Collectively, this study, for the first time, dissected the gene information of *E. annulatus* and revealed the gene expression pattern of *E. annulatus*, providing a valuable foundation for future studies into the molecular biology of *E. annulatus*, which may facilitate the identification of candidate molecular targets relevant to *E. annulatus* monitoring and control.

## Supplementary Information

Additional file 1: Figure S1. Schematic representation of anatomical dissection of adult female *E. annulatus.*

Additional file 2: Table S1. Primers used for qRT-PCR.

Additional file 3: Table S2. Functional annotation of PacBio Iso-Seq transcripts.

Additional file 4: Table S3. BUSCO assessment of the *Eucoleus annulatus* full-length transcriptome.

Additional file 5: Table S4. Unigene transcription factor (TF) prediction.

Additional file 6: Table S5. LncRNA prediction results.

Additional file 7: Table S6. Statistics of Illumina RNA-Seq data for the head, middle, and tail of *E. annulatus*.

Additional file 8: Table S7. Differentially expressed genes identified in the head of *E. annulatus*.

Additional file 9: Table S8. Differentially expressed genes identified in the middle of *E. annulatus*.

Additional file 10: Table S9. Differentially expressed genes identified in the tail of *E. annulatus*.

Additional file 11: Table S10. Complete GO and KEGG enrichment results for Mfuzz Cluster 3 (head-enriched transcripts; corresponding to Fig. 4).

Additional file 12: Table S11. Complete GO and KEGG enrichment results for Mfuzz Cluster 5 (middle-enriched transcripts; corresponding to Fig. 4).

Additional file 13: Table S12. Complete GO and KEGG enrichment results for Mfuzz Cluster 6 and Cluster 7 (tail-enriched transcripts; corresponding to Fig. 4).

## Data Availability

The data supporting the findings of this article are included within the article and its additional files. The raw transcriptomics data have been deposited at China National Center for Bioinformation with accession no. CRA039208.

## Abbreviations

AS: Alternative splicing
BUSCO: Benchmarking Universal Single-Copy Orthologs
CCS: Circular consensus sequence
ceRNA: Competitive endogenous RNA
COG: Clusters of orthologous groups
CPC2: Coding potential calculator 2
CNCI: Coding–noncoding index
DETs: Differentially expressed transcripts
eggNOG: Evolutionary genealogy of genes: Non-supervised Orthologous Groups
FDR: False discovery rate
FL: Full-length
FLNC: Full-length nonchimeric
FPKM: Fragments per kilobase of transcript per million mapped reads
GO: Gene Ontology
ICE: Iterative clustering and error correction
IR: Intron retention
Iso-Seq: Isoform sequencing
KEGG: Kyoto Encyclopedia of Genes and Genomes
KOG: Eukaryotic orthologous groups
lncRNA: Long noncoding RNA
MXE: Mutually exclusive exon
Nr: NCBI non-redundant protein database
OGT: *O*-GlcNAc transferase
PacBio: Pacific Biosciences
PCA: Principal component analysis
Pfam: Protein family database
qRT-PCR: Quantitative real-time polymerase chain reaction
RNA-Seq: RNA sequencing
RQN: RNA quality number
SMRT: Single-molecule real-time
SSR: Simple sequence repeat
TF: Transcription factor
TLR: Toll-like receptor
