# Supplementary figures and images for: Characterization of the full-length transcriptome of female Eucoleus annulatus and comparative transcriptomic analysis of its head, middle, and tail body sections
Source: Parasit Vectors. 2026 Apr 6;19:209. doi: 10.1186/s13071-026-07388-z (PMC13170309; doi:10.1186/s13071-026-07388-z)

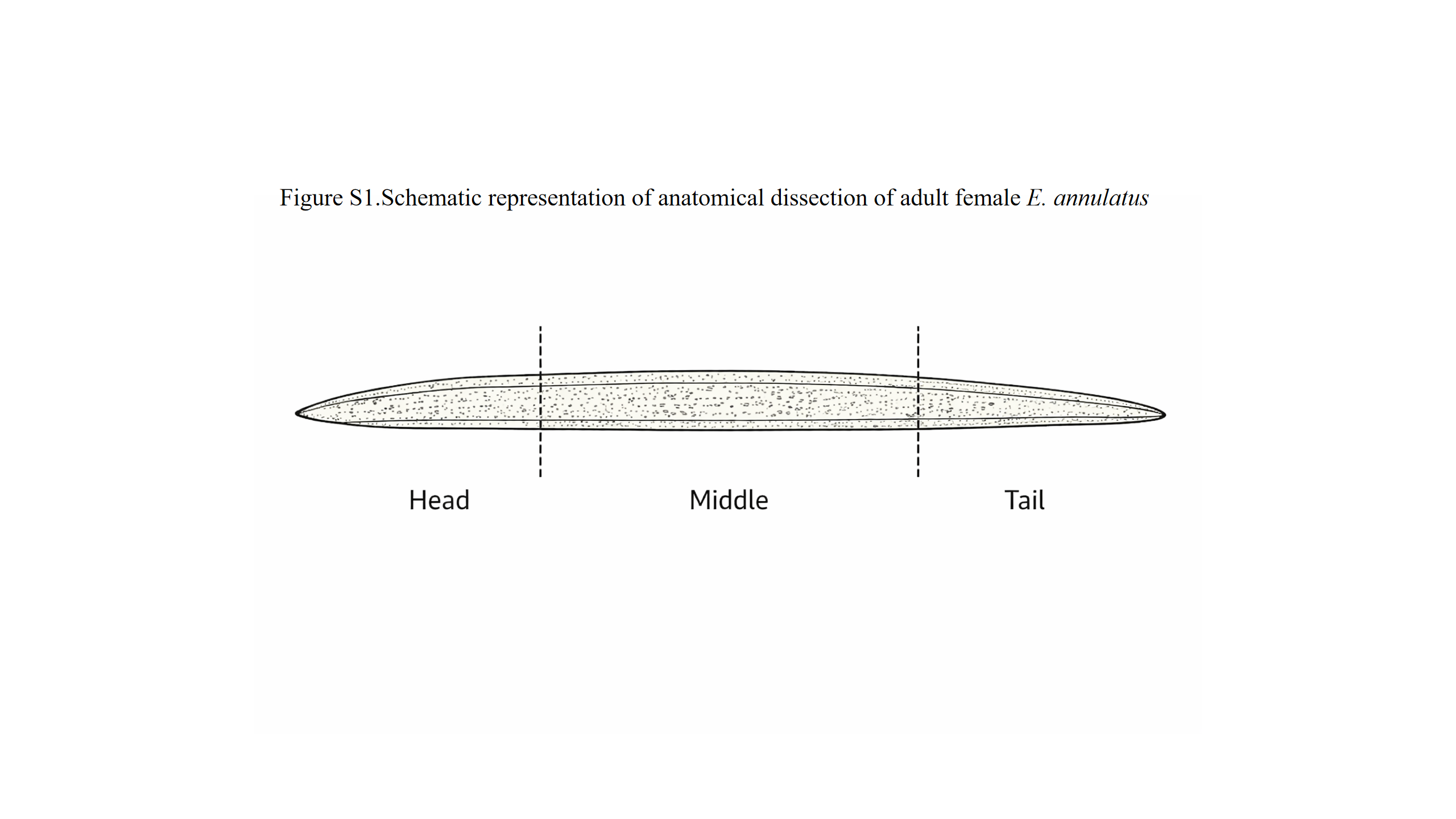

Supplement: Supplementary file 1 — Additional file 1: Figure S1. Schematic representation of anatomical dissection of adult female E. annulatus. [file 13071_2026_7388_MOESM1_ESM.tif]
